# Tissue factor activity on microvesicles from cancer patients

**DOI:** 10.1007/s00432-019-03073-0

**Published:** 2019-11-16

**Authors:** Fanny Ender, Annika Freund, Tabea Quecke, Corinna Steidel, Piet Zamzow, Nikolas von Bubnoff, Frank Gieseler

**Affiliations:** Clinic for Hematology and Oncology, Section Experimental Oncology, University Hospital od Schleswig-Holstein, UKSH, Campus Luebeck, Ratzeburger Allee 160, 23528 Luebeck, Germany

**Keywords:** Extracellular vesicles, Tissue-factor pathway, Progressive cancer

## Abstract

**Purpose:**

The expression of active tissue factor (TF) on the surface of microvesicles (MVs) is essential for the activation of the coagulation system and transduction of the signaling pathways in cancer cells. In its use as a biomarker for cancer-associated venous thromboembolism (VTE), TF has shown high expression variability. As a contribution to this discussion, we present a study investigating plasma samples from patients with various progressive tumors at high risk for VTE.

**Methods:**

Based on our previous study uncovering microvesicles (MVs), the larger ectosome-like extracellular vesicles (EV), as the major source of TF activity in EV preparations, we now determined TF activity on enriched MVs isolated from plasma of cancer patients and compared it with that on MVs from healthy individuals.

**Results:**

We found considerably higher amounts of MVs as well as higher levels of MV-bound TF activities in the plasma of cancer patients. We also show that preparations from plasma of cancer patients have the potency to induce ERK phosphorylation in a human tumor cell line through proteinase-activated receptor two (PAR2) activation.

**Conclusion:**

We suggest that MVs instead of whole EV preparations, and TF activity rather than its antigenic quantification should be used in clinical studies for identifying patients with progressive tumors at high risk for VTE.

**Graphic abstract:**

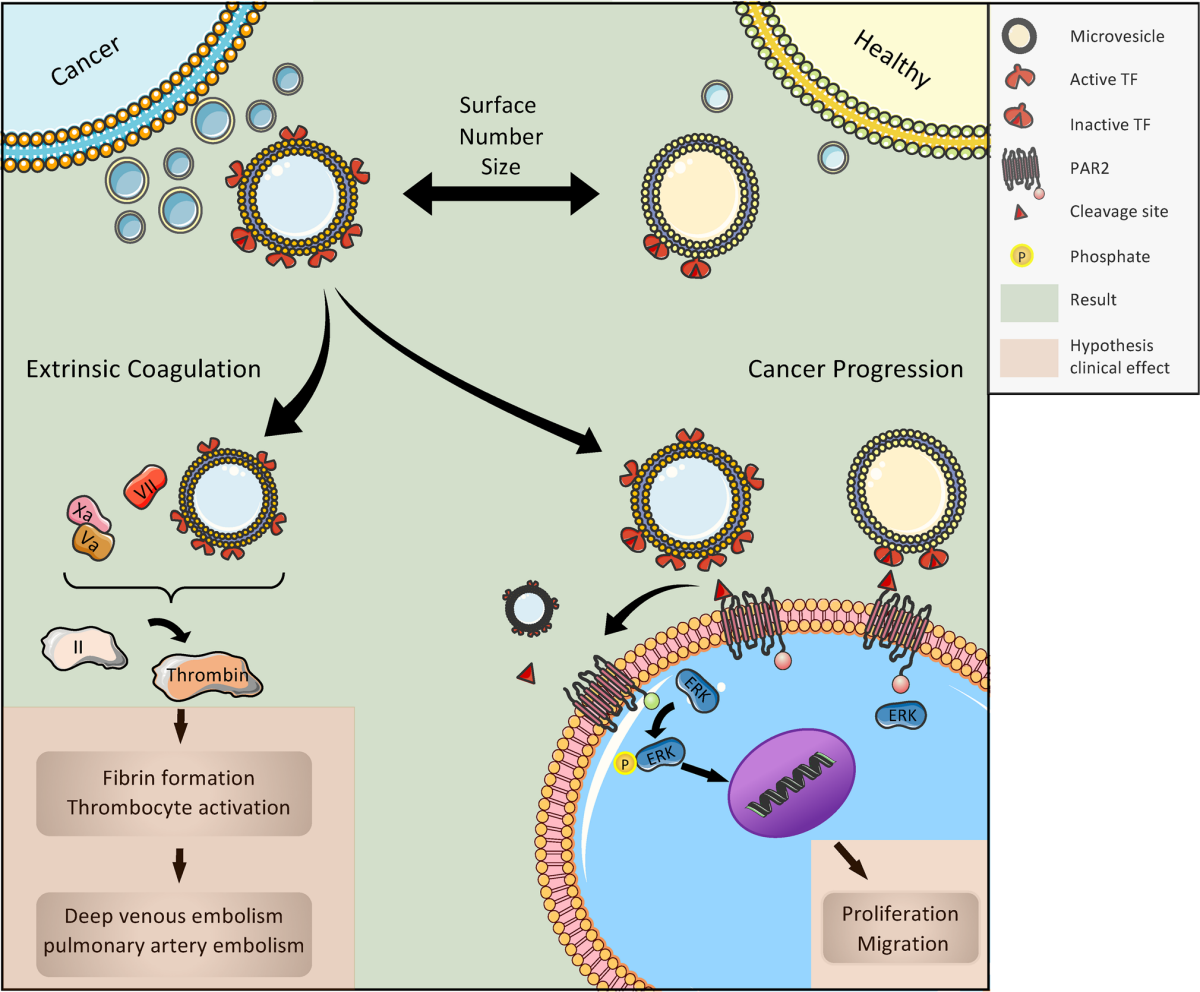

**Electronic supplementary material:**

The online version of this article (10.1007/s00432-019-03073-0) contains supplementary material, which is available to authorized users.

## Introduction

The development of new flow cytometers with resolution capabilities for sub-micron particles has enabled visualization and characterization of small vesicle subpopulations. In principle, two different forms of extracellular vesicles (EVs) can be distinguished: smaller exosomes (30–100 nm) formed intracellularly within multi-vesicular bodies and released after fusion with the plasma membrane, and larger microvesicles (MVs) (50–1000 nm) directly arising by outward budding and fission of the plasma membrane (Van Niel et al. 2018). To date, consensus has not yet emerged on specific markers of EV subtypes. Therefore, the International Society for Extracellular Vesicles has suggested an EV nomenclature that refers to physical characteristics, biochemical composition, or descriptions of conditions or cellular origin (Théry et al. 2018). In this study, we separated EV subsets through sequential centrifugation based on size and refer to larger vesicles as MVs.

Formerly, it was suggested that EVs were a kind of cellular garbage, but now we know that EVs are formed and released by almost every cell in a directed and highly regulated process (Colombo et al. 2014; Yanez-Mo et al. 2015). They can convey intercellular signals; tumor cells, in particular, and also cells of the immune system use EVs to modulate the surrounding microenvironment through targeted signals, such as activation of the proteinase-activated receptor-2 (PAR2)/ERK pathway (Robbins and Morelli 2014).

Tumor cells release EVs that present tissue factor (TF), which is able to systemically activate the extrinsic tenase of the coagulation system through the TF pathway (Tesselaar et al. 2007). As this phenomenon can be observed in vitro by using isolated EVs from tumor cell lines and adding plasmatic coagulation factors, clinical studies aim at correlating tissue factor-positive EVs (TF^+^EVs) with clinical events such as venous thromboembolism (VTE) (Geddings and Mackman 2013). The clinical background is the high rate of VTE, a major life-threatening event, in patients with progressive cancers. As this pathway can be effectively inhibited, e.g. by using low molecular weight heparins such as tinzaparin (Gamperl et al. 2016), the use of surrogate parameters that indicate a high risk of VTE for the individual patient is warranted. Several studies have shown that increased levels of TF^+^EVs correlate with VTE in patients with cancer (Geddings and Mackman 2013; Khorana et al. 2008). Nevertheless, these correlations are not uniformly found over all cancer types (Thaler et al. 2012), and the topic is still under debate (Gardiner et al. 2015). As the underlying principle, namely the systemic activation of the coagulation system by TF^+^EVs, is basically accepted, one of the reasons for the lack of clinical collation in all cancers might be related to the methodologies used in different studies (Gardiner et al. 2015; Thaler et al. 2012). In this context, it has recently been suggested that functional, rather than quantitative assays, based on FXa generation by purified TF^+^EVs may improve sensitivity for predicting thrombotic risk (Graf and Ruf 2018). In the study presented here, we performed functional TF assays using MVs (Gamperl et al. 2016) isolated from the plasma of patients with various progressive cancers and compared the results with those from healthy donors. We have shown before that MVs are the major source of TF activity in EV preparations (Gamperl et al. 2016). The rationale to choose this patient population is their high VTE risk (Donnellan and Khorana, 2017).

## Materials and methods

### Characteristics of healthy donors and cancer patients

All experiments done on human material are in agreement with the Declaration of Helsinki. All volunteers gave informed consent and the study was approved by the Ethics Committee of the University of Luebeck (file number 15–320). Plasma was obtained from 28 healthy donors without any disease and 28 patients suffering from various, advanced and metastatic solid tumors with a high risk to develop VTE. The average age of healthy donors was 29 ± 5 years (60% females, 40% males). The reason for choosing a younger group as negative control is given in the discussion.

The cancer patients in our study were 70 ± 14 years old (40% females, 60% males) and characterized by an advanced tumor stage (stage IV), as tumors had metastasized in 93%, and patients had developed resistance to previous anti-cancer treatment. As patients with progressive cancers, all these belong to the high-risk group for developing VTE (Donnellan and Khorana 2017). The group included patients with cancers of the gastrointestinal tract (50%), genitourinary tract (17%), female breast (10%), respiratory tract (10%), hematologic-immunologic system (4%), sarcoma (3%), neuroendocrine system (3%), and cancers of unknown primary (CUP) (3%).

### Isolation of EVs from plasma of healthy donors and cancer patients

Blood from healthy donors and from patients with advanced stage solid tumors was collected into 7.5 mL lithium-heparin tubes, 2.9 mL citrate tubes and 2.6 mL EDTA tubes (Sarstedt, Nuremberg, Germany). Samples were centrifuged at 470×*g* for 10 min to remove cells and cellular debris. This *“platelet*-*poor plasma”* was kept stored at − 20 °C until used. After thawing, EVs were isolated as described before (Gamperl et al. 2016): samples were centrifuged at 2500×*g* for 15 min at 4 °C, decanted and centrifuged again at 2500×*g* for another 15 min. This low-speed centrifugation protocol is in accordance with the recommendation of the International Society on Hemostasis and Thrombosis (ISTH) and removes cellular debris and larger EVs such as apoptotic bodies and oncosomes (Stagnara et al. 2012). For separation of MVs from smaller exosome-like EVs as well as soluble factors such as interleukins and growth factors, a high-speed centrifugation step at 10,000×*g* for 90 min at 4 °C was performed as described before (Lacroix et al. 2013; Muralidharan-Chari et al. 2009). MV-containing pellets were resuspended in PBS for further analysis.

### MV quantification by high-resolution flow cytometry

Samples were analyzed using the high-resolution flow cytometer *Cytoflex S* (Beckman Coulter, Krefeld, Germany) that enables detection of particles as small as 150 nm in diameter (Spittler 2015). Set-up and configuration were performed as recommended by the manufacturer for the measurement of EVs. Thereby, the violet side scatter (VSSC) 405/10 served as trigger channel. Analysis was performed until 100,000 events were collected for each tube or at least for 3 min. The flow rate was set to 10 μL/min.

### Scanning electron microscopy

Isolated MVs were fixed with glutaraldehyde at a final concentration of 2.5% in filtered PBS and stored at 4 °C until further preparation. After homogenization, 10–20 µL of each sample was placed onto a Thermanox coverslip (Thermo Fisher Scientific, Darmstadt, Germany) and allowed to settle for 90–120 min in a humid chamber to prevent drying. For dehydration, the samples were then placed into solutions with increasing acetone concentration (70–100%) and subsequently fully dried via critical point drying using CO_2_ to avoid shrinkage effects and loss of structure from air-drying. Dehydrated samples were sputtered with gold and analyzed with an EVO LS 15 scanning electron microscope (Zeiss, Germany).

### Determination of TF activity on the surface of MVs

Tenase activity of TF-bearing MVs was determined using the Zymuphen MP-TF Assay (Aniara, West Chester, OH, USA). The wells of the 96-well microplate were coated with a murine, monoclonal anti-human TF antibody, which does not interfere with TF activity. TF-positive MVs within the samples bound to the antibody during an overnight incubation. TF concentration was determined by the potency to activate factor X after the addition of factor VIIa and factor X. The resulting TF/factor VIIa complex cleaved factor X into factor Xa (FXa). In a third step, a specific chromogenic FXa substrate was added, which reacted with FXa leading to a substrate turnover. The absorbance, measured at 405 nm on a photometer, was directly proportional to the amount of TF presented on MVs in the samples. Samples were measured in quadruplicates.

### Cell culture

All cell-based assays were performed using the pancreas carcinoma cell line COLO 357. These cells, originating from a metastasis of a human pancreatic adenocarcinoma, were established and first described by Morgan et al. (Morgan et al. 1980). The cell line grows as an adherent monolayer and has a rather short cell-doubling time of 21 h. It was chosen because of its stable expression of PAR1 and PAR2 and its low spontaneous migratory capacity (Meitner et al. 1983). Cells were cultured under serum-free conditions with 10% panexin (Pan Biotech, Aidenbach, Germany) as serum substitute. We decided to use panexin rather than fetal calf serum, due to the large amount of EVs that can be found in fetal calf serum that would interfere with the experiments (Théry et al. 2006).

### Analysis of MV-induced cell signaling

The potency of TF-bearing MVs to activate the ERK 1/2 signaling pathway via PAR2 was determined using the cell-based human ERK1/2 (Thr202/Tyr204) phosphorylation ELISA (RayBiotech Inc., Norcross, GA, USA). COLO 357 cells were seeded into the wells of a 96-well microtiter plate 24 h prior to use. Three hours before stimulation with MVs, cells were set to starving medium containing only 1% panexin. Thereafter, cells were incubated with isolated plasma MVs for 10 min. After stimulation, the assay was performed according to the manufacturer’s instructions: briefly, cells were fixed, quenched and blocked before incubation with a murine monoclonal anti-human ERK1/2 antibody or a murine monoclonal anti-human phosphoERK1/2 antibody. Staining was performed using a secondary anti-mouse IgG antibody coupled to horseradish peroxidase (HRP). After administration of the HRP’s substrate TMB, the substrate turnover, measured at 450 nm, was proportional to the amount of phosphorylated or unphosphorylated ERK.

### Statistical analysis

All experiments were performed at least in triplicate. Results are shown as individual data points and were analyzed using mean and standard deviation. To test the statistical difference between groups, Student’s *t* test for unequal variances was used. We further evaluated correlations between analyzed parameters by computing Pearson’s correlation coefficient (Microsoft Excel for Mac 16.16.8, GraphPad Prism 8.1.1, San Diego, CA, USA).

## Results

### High number of MVs in the plasma of patients with metastasized tumors

**Fig. 1:**
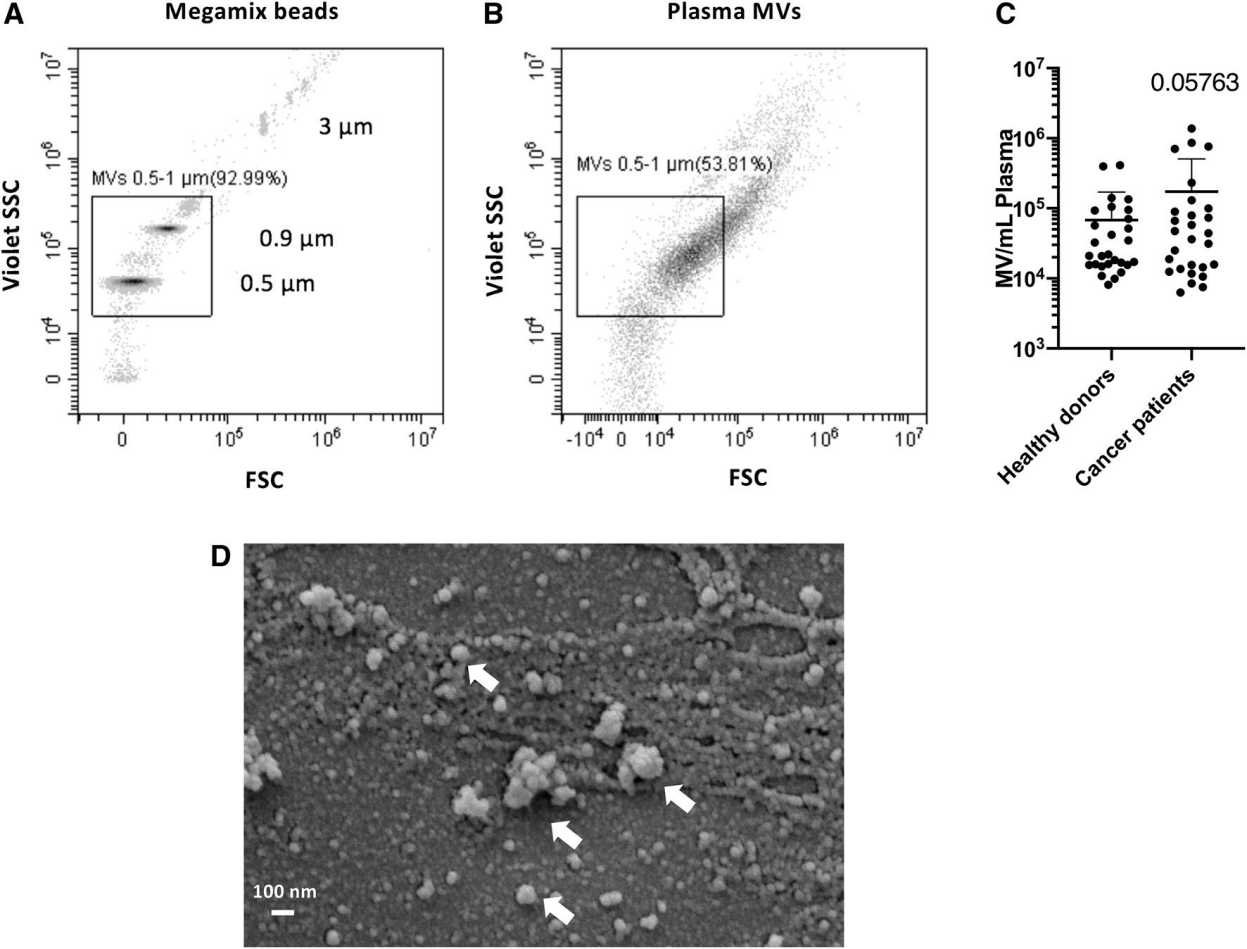
Microvesicles (MVs) in the plasma of patients with progressive cancers and in healthy donors in high-resolution flow cytometry. **a** Localization of Megamix beads for size calibration. Bead populations with distinct diameters (0.5, 0.9, 3 µm) are detectable through *violet side scatter triggering* and presented as density plot. **b** Plasma MVs identified by high-resolution flow cytometry. Events within the size range of 0.5-1 µm were defined as MVs. **c** Quantification of plasma MVs in the size range of 0.5-1 µm from healthy donors and cancer patients. Shown is the mean ± SD; *n* = 28. Due to the high inter-individual variance, especially in cancer patients, the differences did not reach statistical significance (*p* = 0.05763). **d** Characterization of extracellular vesicles by SEM

Plasma MVs from healthy donors and cancer patients were detected and quantified by high-resolution flow cytometry. As a size reference, we used Megamix beads (Biocytex, Marseille, France), which comprise populations with different diameters including 0.5, 0.9 and 3 µm (Fig. 1a). For the identification of MVs in the size range of 0.5–1 µm, we applied a respective size gate based on the Megamix beads and counted all events within that gate as MVs (Fig. 1b). Using flow cytometry (Fig. 1b) and scanning electron microscopy (Fig. 1d), we could confirm a selective enrichment of larger vesicles heterogeneous in size by our sequential centrifugation protocol. The total count of plasma MVs from healthy donors and cancer patients is shown in Fig. 1c. In healthy donors, we found an MV concentration of 0.68 × 10^5^ ± 1.03 × 10^5^ MV/mL plasma; in patient plasma, the MV concentration was 1.73 × 10^5^ ± 3.33 × 10^5^. We also found a very high inter-individual difference, especially in cancer patients with a range of two log steps, which prevented statistical significance.

### TF activity levels of plasma MVs from cancer patients are significantly higher as compared to healthy donors

**Fig. 2:**
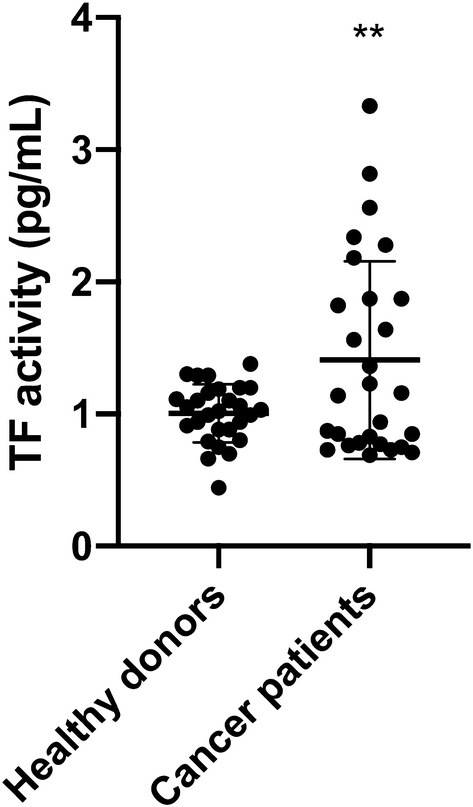
Tissue factor (TF) activity of plasma MVs from healthy donors and cancer patients. Shown is the mean ± SD; *n* = 28; **p < 0.01. Note the high inter-individual variance in cancer patients

The presentation of TF is a clinically and functionally relevant characteristic of MVs, as it defines them as potent activators of the extrinsic coagulation system and other cellular signaling pathways such as PAR2/ERK. However, if TF antigen is detected on the surface of MVs, it does not necessarily mean that it is functionally active as previously discussed (Graf and Ruf 2018). Therefore, we measured tenase activity of TF-bearing MVs using the Zymuphen MP-TF assay as described above. Plasma MVs from healthy donors had a TF (with tenase activity) concentration of 1.01 ± 0.22 pg/mL, whereas plasma MVs from patients with advanced tumors showed a significantly higher level of TF activity of 1.41 ± 0.75 pg/mL (*p* < 0.01) (Fig. 2).

### TF-bearing MVs from cancer patients are potent inducers of the ERK signaling pathway in tumor cells

**Fig. 3:**
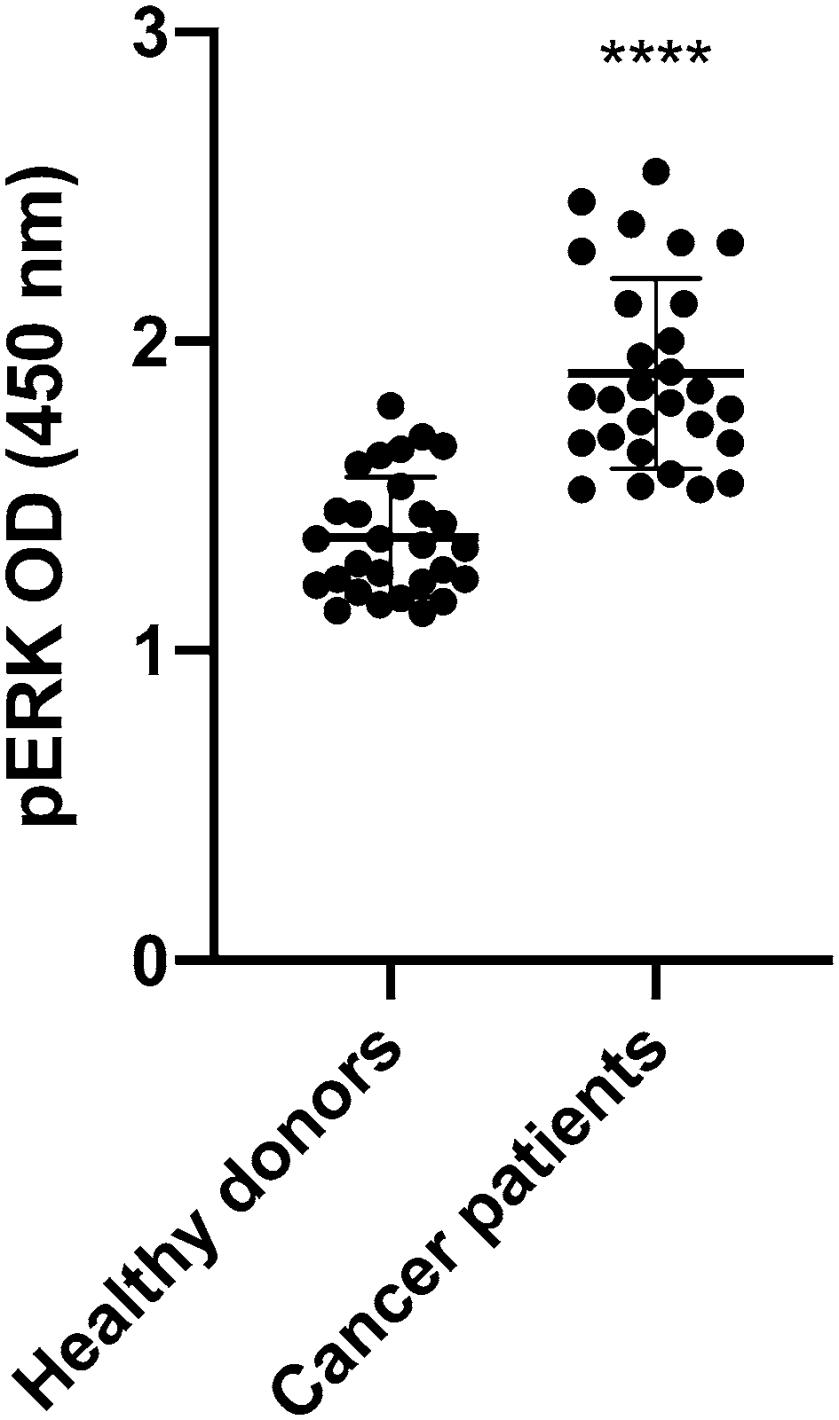
Plasma**-**MV mediated induction of ERK phosphorylation in COLO 357 human pancreas carcinoma cells after 10 min of incubation. Shown is the mean ± SD; *n* = 28; ****p < 0.0001

The presentation of TF on the surface of MVs is not only important for the activation of extrinsic coagulation but also for that of surface receptors, such as PAR2, on target cells, which leads to the induction of intracellular signaling and subsequently influences cellular functions (Geddings and Mackman 2013). We investigated the potential of plasma MVs to induce ERK phosphorylation in the human pancreatic carcinoma cell line COLO 357. Plasma MVs from cancer patients triggered significantly higher ERK phosphorylation as compared to MVs from healthy donors. Of note, plasma MVs induced ERK phosphorylation in the same range as a specific PAR2 agonist (Fig. 3; controls are shown in supplemental Fig. 1).

### Direct correlation between TF activity and ERK phosphorylation of MVs from healthy donors and cancer patients

**Fig. 4:**
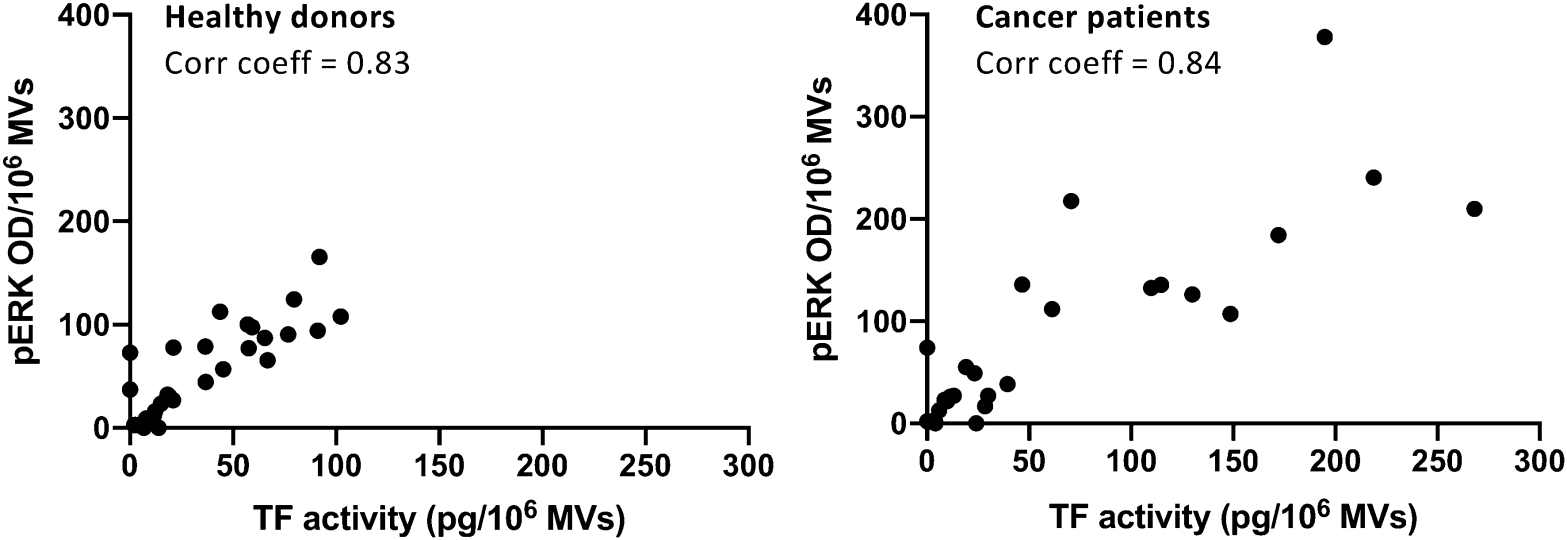
Direct correlations between TF activity and ERK phosphorylation of MVs isolated from healthy donors (left) and cancer patients (right). TF activity and ERK phosphorylation was normalized to 10^6^ MVs. Pearson’s correlation coefficients > 0.8

To determine functional properties on MV levels, we normalized TF activity and ERK phosphorylation to 10^6^ MVs and found a linear correlation between pERK and TF activity with a correlation coefficient > 0.8 for plasma MVs of both healthy donors and cancer patients (Fig. 4). Of note, the magnitude of a basal ERK phosphorylation induced by plasma MVs is comparable to that induced by a specific PAR2 agonist (supplemental Fig. 1) suggesting that cellular activation occurs via an the PAR2/ERK signaling axis as previously suggested (Gieseler et al. 2013; Graf and Ruf 2018; Ruf et al. 2011).

## Discussion

TF^+^EVs are a major source of blood-borne TF in the context of inflammation and malignancy, thus being potentially responsible for the activation of the coagulation system and subsequently high VTE rate in cancer patients (Date, Eitan et al. 2017). Although clinical studies have shown that increased levels of TF^+^EVs in principle correlate with venous thrombosis in patients with cancer (Geddings and Mackman 2013; Khorana et al. 2008), these correlations are not uniformly found over all cancer types (Thaler et al. 2012). In fact, huge variances of TF^+^EV numbers as well as variations of VTE rates are found in clinical studies, indicating pathophysiological differences between different forms of cancer and patients with comorbidities as well as medication affecting EVs. Cellular membrane expression of TF has been shown on the majority of tumors and it has been linked to the promotion of hematogenous metastasis (Mueller et al. 1992) and the balance of angiogenic and antiangiogenic properties of tumor cells (Zhang et al. 1994). As MVs belong to the EV sub-group of ectosomes, they are formed by outward budding and fission of the plasma membrane, thus representing the membrane composition of their parent cells (Van Niel et al. 2018). Therefore, in principle, they could be used as liquid biopsies in the diagnosis of cancer (Ender et al. 2019; Mathai et al. 2019).

A number of different co-factors such as protein disulfide isomerase (PDI) or the co-exposure of phosphatidylserine on the outer layer of cell membrane modulate TF activity (Popescu et al. 2010). In addition, it has been proposed that the expression of TF isomers, namely encrypted or decrypted TF, with only the latter significantly contributing to extrinsic tenase activity (TF-FVIIa), might be responsible for the variations of TF activity (Graf and Ruf 2018). Therefore, in this study we performed assays that determined TF activity rather than TF concentration on MVs (larger ectosome-like vesicles) isolated from patients with progressive cancers. Using sequential centrifugation steps including high-speed centrifugation, we enriched MVs to achieve more uniform results. In order to judge the results, we have compared them to those from healthy donors without evidence of thrombosis. For this study, we decided to choose a true negative population of younger age, with no signs of any disease including thrombosis, and no medication, since age-matched controls would presumably suffer from co-morbidities, risk factors for age-related diseases and poly-medication. The best control group would have been the same patient before and after cancer diagnosis, which is not possible. The issue with people at higher age is heterogeneity regarding co-morbidities potentially influencing the number and structure of EVs (Extermann 2000).

Plasma EVs show age-related changes that affect not only the number but also the structure and content of the vesicles. Although senescent cells release more EVs (Robbins and Morelli 2014), plasma EV concentration decreases with advancing age, probably because EVs from older individuals are more readily internalized by B cells (Eitan et al. 2017). EVs activate both monocytes and B cells, and activation of B cells by lipopolysaccharide (LPS) enhances EV internalization (Eitan et al. 2017). As our results showed more EVs in older patients (Fig. 1c), we assume that this effect is disease related and not just a matter of aging. Using flow cytometry or SEM for the characterization of plasma MVs, we could show that sequential centrifugation leads to selective enrichment of larger vesicles heterogeneous in size. Notably, determining the actual size of plasma EV preparations after high-speed centrifugation led to different results depending on the method that was used. While we identified particle sizes up to 3 µm by flow cytometry and the use of Megamix beads with defined size populations (Fig. 1b), we could identify particles within a size range of 50–500 nm by SEM (Fig. 1d). This discrepancy can be linked to the properties of the polystyrene beads we used for size calibration. We are aware that the refractive index of these beads differs from those of cells and EVs (Chandler et al. 2011; Mullier et al. 2011; Robbins and Morelli 2014). Lipid-based vesicles have a lower refractive index and hence, scatter much less light than polystyrene beads, which might influence size determination. Importantly, we found that Megamix bead-based size determination of EV preparations by flow cytometry seems to overestimate particle sizes.

Although the increased MV levels in the plasma of patients with progressive cancers did not reach the level of statistical significance when compared to healthy donors, the inter-individual variance of MV levels was much higher in cancer patients than in healthy donors overlapping two log-ranges (Fig. 1c). This was also the case for TF activity, which was significantly higher in MVs from the plasma of cancer patients as compared to healthy donors (Fig. 2). These results reflect our analysis of a real-life group of patients treated in our hospital. Given our choice of a control group of healthy young donors, the observed variances are not surprising. These observations are per se remarkable but obviously not sufficient to explain the difference in VTE rates in different cancers. One of the factors that increase the release of EVs is tumor cell stimulation with inflammatory cytokines and endotoxin (Yamamoto et al. 2015). Hence, determining the concentration of EVs might be a promising tool in clinical studies to determine the VTE risk of patients. To show that TF^+^MVs are biologically active, we performed ERK phosphorylation assays using human pancreatic carcinoma cells COLO 357. Although the MAPK/ERK pathway can be activated by several stimuli, it is one of the elemental signaling systems that controls fundamental cellular processes as proliferation, differentiation, survival and apoptosis (Sun et al. 2015). In Fig. 3, it is shown that MVs isolated from cancer patients have a significant higher potency to induce cellular ERK phosphorylation as plasma MVs from healthy donors. On the MV level, we found a direct correlation of TF activity and ERK phosphorylation (Fig. 4), which points to the decisive role of TF activity for the cellular effects of plasma MVs in cancer patients. Using sequential centrifugation for EV purification, we got rid of soluble plasma factors such as cytokines and endotoxin. Thus, plasma MVs resuspended from pellets after high-speed centrifugation are responsible for the described cellular effects.

## Conclusions

The experiments were performed to determine if TF^+^EVs can be used as a surrogate parameter for patients at high risk for VTE. We have shown before that larger MVs are the major source of TF activity in EV preparations (Gamperl et al. 2016). Here we show that patients with progressive tumors being at high risk for VTE have indeed more MVs with significantly higher TF activity. With regard to the high inter-individual variance in the group of older cancer patients, we do not think that TF^+^EVs can be used as a marker for all different types of cancers. Nevertheless, TF^+^EVs can be a surrogate parameter for the risk of developing cancer-associated VTE for individual patients and might indicate the use of low molecular weight heparins with the potency to release TFPI, such as tinzaparin. This effect might be especially beneficial as TF^+^EVs not only activate the coagulation system but also have migration-inducing effects on PAR2-expressing cancer cells (Ender et al. 2019; Gamperl et al. 2016; Gieseler et al. 2013).

## Electronic supplementary material

Supplemental figure 1: MV induced PAR2/ERK signaling in COLO 357 tumor cells. Incubation of pancreatic carcinoma COLO 357 cells with plasma MVs for 10 min induced ERK phosphorylation in the same range as a specific PAR agonist. Cell culture medium and PAR2 agonist SLIGKV-NH2 (100 µM) served as negative and positive control, respectively. Shown is the mean ± SD; n=4; *** p < 0.001; **** p < 0.0001.

Below is the link to the electronic supplementary material.

Supplementary material 1 (TIFF 880 kb)

## Abbreviations

TF: Tissue factor
MVs: Microvesicles
VTE: Venous thromboembolism
EVs: Extracellular vesicles
PAR2: Proteinase-activated receptor 2
SEM: Scanning electron microscopy
TFPI: Tissue factor pathway inhibitor
