# Supplementary figures and images for: Tissue factor activity on microvesicles from cancer patients
Source: J Cancer Res Clin Oncol. 2019 Nov 16;146(2):467–75. doi: 10.1007/s00432-019-03073-0 (PMC6985086; doi:10.1007/s00432-019-03073-0)

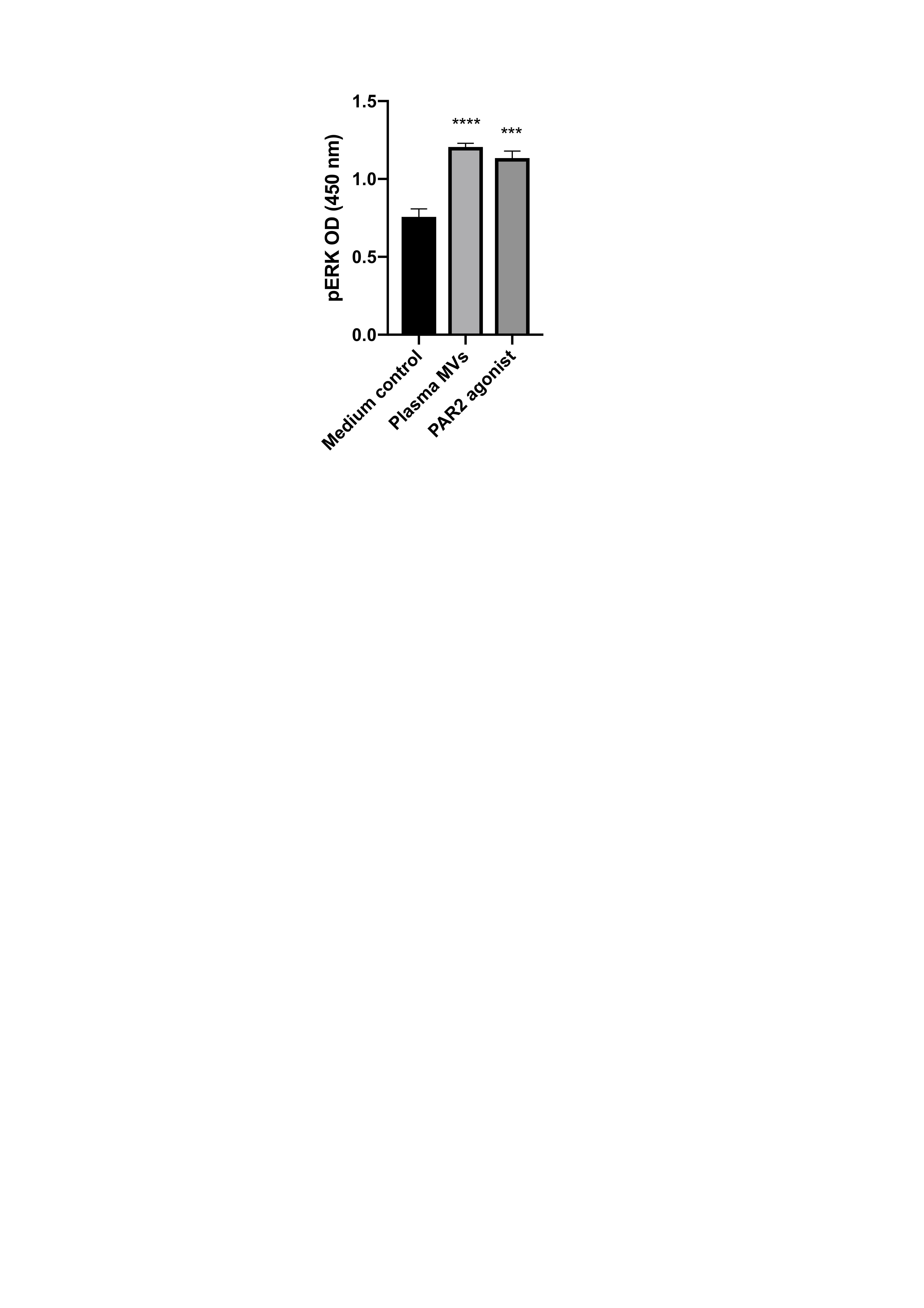

Supplement: Supplementary file 1 — Supplementary material 1 (TIFF 880 kb) [file 432_2019_3073_MOESM1_ESM.tiff]
